# Investigating Colonization of the Healthy Adult Gastrointestinal Tract by Fungi

**DOI:** 10.1128/mSphere.00092-18

**Published:** 2018-03-28

**Authors:** Thomas A. Auchtung, Tatiana Y. Fofanova, Christopher J. Stewart, Andrea K. Nash, Matthew C. Wong, Jonathan R. Gesell, Jennifer M. Auchtung, Nadim J. Ajami, Joseph F. Petrosino

**Affiliations:** a Alkek Center for Metagenomics and Microbiome Research, Department of Molecular Virology and Microbiology, Baylor College of Medicine, Houston, Texas, USA; Carnegie Mellon University

**Keywords:** colonization, diet, fungi, gastrointestinal tract, mycobiome, saliva

## Abstract

A wide diversity of fungi have been detected in the human gastrointestinal (GI) tract with the potential to provide or influence important functions. However, many of the fungi most commonly detected in stool samples are also present in food or the oral cavity. Therefore, to recognize which gut fungi are likely to have a sustained influence on human health, there is a need to separate transient members of the GI tract from true colonizers. To identify colonizing fungi, the eukaryotic rRNA operon’s second internal transcribed spacer (ITS2) was sequenced from the stool, saliva, and food of healthy adults following consumption of different controlled diets. Unlike most bacterial 16S rRNA genes, the only fungal ITS2 operational taxonomic units (OTUs) detected in stool DNA across multiple diets were also present in saliva and/or food. Additional analyses, including culture-based approaches and sequencing of the 18S rRNA gene, ITS2 cDNA, and DNA extracted using alternative methods, failed to detect additional fungi. Two abundant fungi, Saccharomyces cerevisiae and Candida albicans, were examined further in healthy volunteers. *Saccharomyces* became undetectable in stool when a S. cerevisiae-free diet was consumed, and the levels of C. albicans in stool were dramatically reduced by more frequent cleaning of teeth. Extremely low fungal abundance, the inability of fungi to grow under conditions mimicking the distal gut, and evidence from analysis of other public datasets further support the hypothesis that fungi do not routinely colonize the GI tracts of healthy adults.

**IMPORTANCE** We sought to identify the fungi that colonize healthy GI tracts and that have a sustained influence on the diverse functions of the gut microbiome. Instead, we found that all fungi in the stool of healthy volunteers could be explained by their presence in oral and dietary sources and that our results, together with those from other analyses, support the model that there is little or no gastrointestinal colonization by fungi. This may be due to Westernization, primate evolution, fungal ecology, and/or the strong defenses of a healthy immune system. Importantly, fungal colonization of the GI tract may often be indicative of disease. As fungi can cause serious infections in immunocompromised individuals and are found at increased abundance in multiple disorders of the GI tract, understanding normal fungal colonization is essential for proper treatment and prevention of fungal pathogenesis.

## Abstract (precis)

We sought to identify the fungi that colonize healthy GI tracts and that have a sustained influence on the diverse functions of the gut microbiome. Instead, we found that all fungi in the stool of healthy volunteers could be explained by their presence in oral and dietary sources and that our results, together with those from other analyses, support the model that there is little or no gastrointestinal colonization by fungi. This may be due to Westernization, primate evolution, fungal ecology, and/or the strong defenses of a healthy immune system. Importantly, fungal colonization of the GI tract may often be indicative of disease. As fungi can cause serious infections in immunocompromised individuals and are found at increased abundance in multiple disorders of the GI tract, understanding normal fungal colonization is essential for proper treatment and prevention of fungal pathogenesis.

## INTRODUCTION

There are fungi in the gastrointestinal (GI) tracts of all healthy people (1), where they can impact the immune system (2) and produce secondary metabolites that influence health (3, 4). One fungal strain, Saccharomyces cerevisiae var. boulardii, is also commonly used as a probiotic to treat gastrointestinal disorders (5). However, fungi in the GI tract have been associated with numerous diseases based on case-control studies (see, e.g., reference 6) and may also contribute to the millions of invasive fungal infections that occur in immunocompromised individuals every year (7). Considering that ~0.01% to 0.1% of metagenomic reads from adult stool samples have mapped to fungal species (8, 9) and that fungal genomes are ~3 to 10 times larger than bacterial genomes, it is possible to estimate that of the >10^13^ microorganisms in the GI tract (10), about a billion compose what is often referred to as the gut mycobiota or mycobiome (11).

A recurring issue in studies of fungi in the GI tract is how many of the detected fungi are true colonizers in contrast to those transiently passing through the GI tract from other sources such as food, the environment, or the nasal or oral cavities (12). Unlike bacteria, archaea, and some protists, the fungi detected in stool by ribosomal internal transcribed spacer (ITS) amplification and sequencing typically have little longitudinal stability across samples from a single person (1). Most commonly detected fungal genera have representatives present in food and/or the oral cavity (13–15), and some are known to increase in abundance in stool following consumption of certain foods (16). Therefore, any fungal commensals possibly colonizing the GI tract may be obscured in experiments that do not control for the presence of transient fungi.

We sought to differentiate transiently present fungal species from those that colonize the GI tract. However, our controlled-diet and dental hygiene experiments suggested that all of the fungi detected were transiently associated and therefore that possibly no fungi had colonized the GI tracts of the individuals examined. Analyses of large public gut microbiome data sets and the failure of fungi to grow under gut-simulating conditions lend growing evidence to support the hypothesis that fungi are excluded from colonizing the GI tracts of most healthy Western adults.

## RESULTS

### Examining patterns in fungi detected in the stool of Human Microbiome Project volunteers.

**FIG 1:**
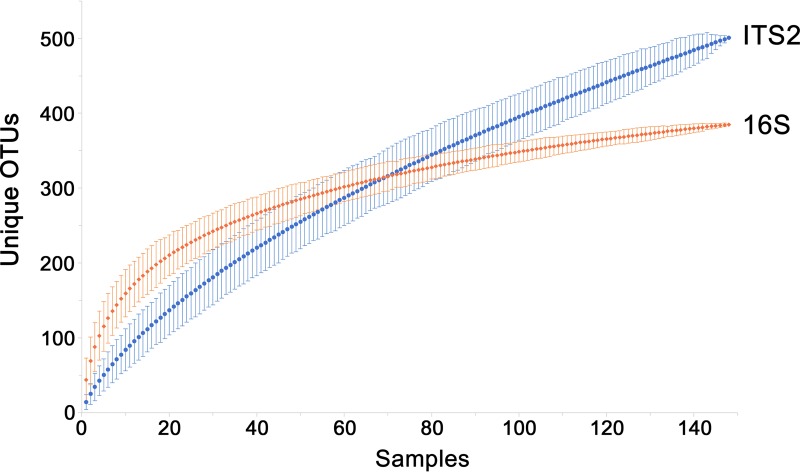
Comparison of community fungal and bacterial/archaeal diversity levels. Numbers of unique ITS2 (blue circles) or 16S rRNA gene (orange diamonds) OTUs in 148 Human Microbiome Project samples (from 100 volunteers) that had been rarefied to 1,000 reads for both regions were determined. Plotted data represent the mean number of OTUs detected at the indicated number of samples when sample order was randomized over 1,000 iterations. The error bars represent the 95% confidence intervals. The ITS2 data most closely fit the power function *y* = 17.34*x*^0.68^ (*R*^2^ = 0.998), whereas the 16S rRNA gene data most closely fit the logarithmic equation *y* = 79.03ln(*x*) −17.84 (*R*^2^ = 0.988).

Because the environment of the GI tract limits colonization to certain organisms, with sufficient sampling, a large portion of the total diversity of colonizers can be identified and the detection of new organisms plateaus. In contrast, transient populations would not be required to survive in the gut and therefore would not have the same restrictions on diversity. The fungi present in stool samples of healthy adults in the Human Microbiome Project (HMP) cohort were previously examined through amplification and sequencing of the eukaryotic rRNA operon’s second internal transcribed spacer (ITS2) (1). We examined the rate at which new fungal ITS2 operational taxonomic units (OTUs) could be detected in HMP stool samples and compared this to the rate of detection of new 16S rRNA gene OTUs using all samples that had at least 1,000 reads from each analysis (148 shared samples from 100 volunteers). Sequences from both regions were clustered into OTUs at 97% identity, which is roughly species level (17). For individual samples, the number of unique ITS2 OTUs was typically lower than that of 16S rRNA gene OTUs. However, when at least 70 samples were examined, ITS2 diversity surpassed that of 16S rRNA genes (Fig. 1). When all 148 samples were included, there were 501 ITS2 OTUs, compared to 385 16S rRNA gene OTUs. The addition of new OTUs most closely follows a power function for ITS2 and a logarithmic curve for 16S rRNA genes. This difference in the rates of identifying new OTUs is consistent with the possibility that most of the detected fungi were ultimately derived from the consumption of different foods, which contain, as a whole, more unique microorganisms than those that may be colonizing the GI tract.

We further analyzed the data to identify the specific fungal taxa consistently present over time. There were 42 volunteers with three longitudinal samples containing at least 1,180 (mean, 54,880) nonplant ITS2 reads each. Although there was a mean of 10.3 (range, 2 to 32) ITS2 OTUs present at ≥10 reads/sample, only OTUs representing Saccharomyces cerevisiae (30 volunteers) and Malassezia restricta (19 volunteers) were present at all sampling points for two or more volunteers (see Table S1 at https://github.com/auchtung/Auchtung2018). However, it is possible that additional, low-abundance fungi were present but obscured by the various fungal species found associated with foods and that they could be discovered by examining the stool of volunteers that consumed controlled diets of known fungal composition.

### Examining fungi in the stool of volunteers across different controlled diets.

To identify fungi colonizing the human GI tract, each of four healthy Western adult volunteers (volunteer 1 [V1] to V4) of diverse geographical and dietary backgrounds consumed the same sequence of four predefined diets of nuts and dried berries (A); beans, rice, and corn (B); pasta and marinara sauce (C); and an egg, sausage, and cheese sandwich and peas (D) (see Table S2A and B at https://github.com/auchtung/Auchtung2018). The diets were selected for their representation of major food groups, their relatively low fungal load, and their simplicity, which allowed all components to be sequenced to identify the fungi present. Saliva samples were collected throughout, and the second stool sample seen to contain food or dye from the particular diet was collected (the time of collection differed among the samples but was typically 2 days after the volunteer commenced the diet). All volunteers also provided stool and saliva samples on a day when they consumed their regular (uncontrolled) diet. Concurrently, a fifth healthy volunteer (V5) on an uncontrolled diet also donated stool samples. Fungi present in stool, saliva, and controlled-diet components were examined by sequencing the ITS2 region from extracted DNA. Orthogonal approaches were taken to ensure that no fungi in stool went undetected, including amplifying and sequencing ITS2 cDNA from reverse-transcribed RNA and 18S rRNA genes from DNA, examining fungi in DNA extracted by different methods, and culturing viable fungi. For comparison, the bacterial and archaeal community was examined by 16S rRNA gene sequencing as necessary.

The abundance of ITS2 and 16S rRNA genes in DNA extracted from stool was examined by quantitative PCR. ITS2 copies were 680 to 7,600 (mean, 3,000) times less abundant than 16S rRNA genes (see Fig. S1 at https://github.com/auchtung/Auchtung2018). The ITS2 region was also amplified from stool DNA for sequencing on an Illumina MiSeq platform (2 × 300 bp). A high fraction of sequence reads from all controlled-diet volunteers matched plant sequences (see Table S2C and Fig. S2 at https://github.com/auchtung/Auchtung2018; V1 = 68%, V2 = 42%, V3 = 94%, and V4 = 91%). These matched either foods consumed in the controlled diets (see Table S2D at https://github.com/auchtung/Auchtung2018; *Prunus* almonds in diet A and *Pisum* peas in diet D) or foods previously consumed by the volunteers, particularly those associated with seeds (*Rubus* raspberries in V1, *Linum* flax in V2, *Amaranthus* of unknown origin in V3, and *Psidium* guava and *Solanum* tomatoes in V4). Volunteers 3 and 4 had no memory of having consumed amaranth or guava within 2 weeks of the study, suggesting that they represented a minor component of the fecal material and were detected only because of the small amount of fungi present and the slow transit times of seeds. The relative amount of plant reads was greater than that observed in V5 and HMP volunteers (both 16%), possibly due to the controlled diets being relatively low in fungi and high in plants that amplify well with the primers employed.

**FIG 2:**
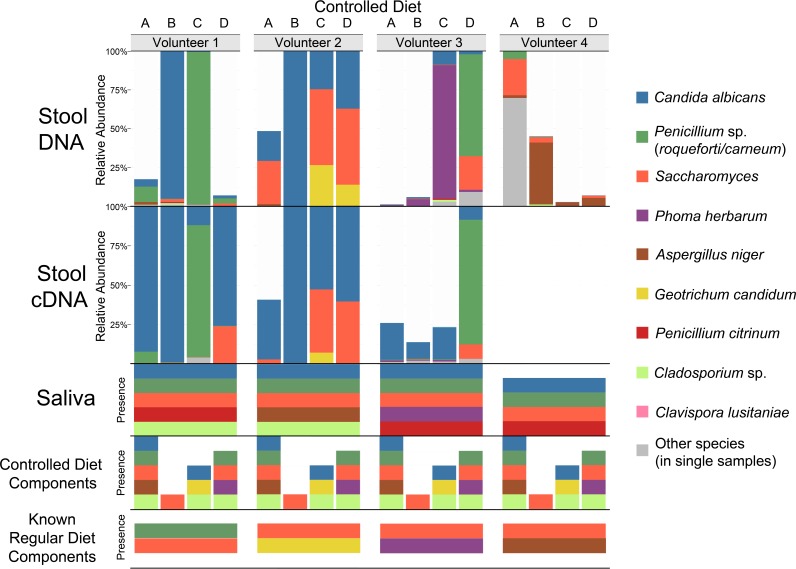
Relative abundances of fungal taxa detected by ITS2 DNA and cDNA analysis of stool samples from volunteers 1 to 4 following controlled diets A to D and the presence of those taxa in the saliva of the volunteers, components of the controlled diets, and known components of the volunteers’ regular diets. Stool DNA, stool cDNA, saliva, and controlled-diet samples had median read values of 24,655, 10,527, 18,540, and 18,500, respectively. For stool, the relative abundances for triplicate samples were averaged and sequences mapping to plants were removed. Samples with >1,000 reads remaining were rarefied to 1,000 reads. For saliva, there were 10 to 12 samples analyzed per volunteer that were collected across the diets. For controlled-diet components, two samples were analyzed (typically one whole sample and one sample rinse). A species was considered present if detected at ≥10 reads in any replicate of a sample. The species detected from stool samples of >1 diet of a volunteer are indicated with coloring. Regular diet components were those foods volunteers reported typically eating.

When plant reads were removed from the ITS2 DNA data, the volunteers had high levels of fungi that corresponded to species abundant in their saliva, to species abundant in foods that the volunteers reported frequently consuming during their regular diet, or to species abundant in foods that were components of the experimental diet: Candida albicans (saliva) or *Penicillium* sp. (blue cheese) in V1, C. albicans (saliva) and/or *Saccharomyces* (multiple foods) in V2, Phoma herbarum (saliva and beer) in V3, and Aspergillus niger (nut mix) (18) in V4 (Fig. 2; see also Table S2D and E at https://github.com/auchtung/Auchtung2018). In addition, all volunteers had detectable levels of A. niger in diet A (multiple nuts and fruits) and *Saccharomyces* in diets B (corn) and D (bread).

Considering the unrarefied ITS2 DNA sequences from stool of the four controlled diets, there were 60 fungal OTUs in total but just 10 OTUs that were found in two or more stool samples from any volunteer (see Table S2C at https://github.com/auchtung/Auchtung2018; an OTU was considered present if there were at least 10 reads in any replicate of a sample). Of those 10 OTUs, 8 were also detected in saliva, and 8 were also detected in food. The 10 OTUs included those at the highest relative abundance (mentioned above): C. albicans, *Penicillium* sp., *Saccharomyces*, P. herbarum, and A. niger, plus Geotrichum candidum (other than in controlled-diet C, where it was detected in the pasta, it was detected only in V2 diet D stool samples, possibly due to the presence of cheese in the regular diet consumed by V2), *Cladosporium* sp. (a common mold in the environment, detected at low levels from the volunteers and from seven foods across three controlled diets), Penicillium citrinum (not detected in food but present in all four volunteers’ saliva samples), and Xanthophyllomyces dendrorhous (extraction or PCR contaminant detected at very low levels in all sample types, including controls). Clavispora lusitaniae was the only OTU that was detected in multiple stool samples of a volunteer but not in food or their saliva. However, its level narrowly surpassed the defined limit of detection in stool (12 and 47 reads in one of three replicates of two different V2 samples) and fell just short of the limit of detection in food (8 reads in blueberries). C. lusitaniae is commonly found in the environment and was also detected in dust from a shelf in the laboratory where the samples were processed (see Table S2F at https://github.com/auchtung/Auchtung2018). Although it could have been colonizing V2, it was more likely detected by chance due to low-level contamination of food that the volunteer ate or during sample processing. Unlike ITS2, 138 of the 162 total 16S rRNA gene OTUs were detected in two or more stool samples from any volunteer, with only 6/138 also present in saliva (see Fig. S3 and Table S2G and H at https://github.com/auchtung/Auchtung2018). The 16S rRNA genes in food were not analyzed.

**FIG 3:**
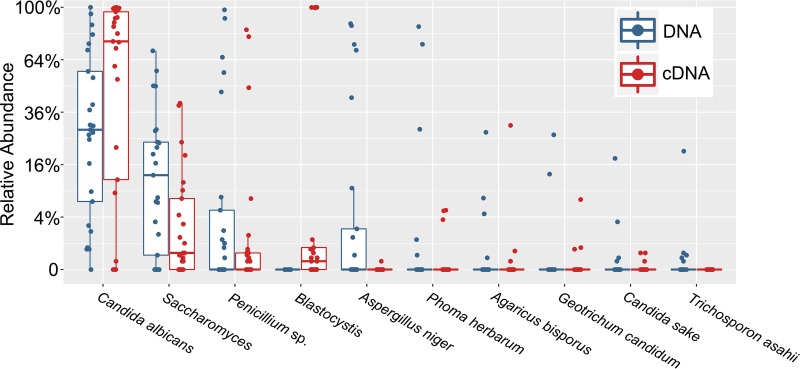
Relative abundances of microeukaryotic taxa detected by ITS2 analysis of DNA or cDNA from stool. Taxonomic differences between ITS2 DNA (blue) and cDNA (red) analyses following normalization to an equal number of microeukaryotic reads/sample were determined. Twenty-five samples from four volunteers on four controlled diets and one uncontrolled diet and from one volunteer on five uncontrolled diets were analyzed. Boxes contain the interquartile range, with the center line denoting the median relative abundance.

Due to the reduced stability of free RNA compared to DNA, ITS2 cDNA has the potential to reveal more about the active community. The method has associated caveats such as that rRNA is relatively stable, the amount of rRNA produced varies across species, and even dormant cells can maintain high numbers of ribosomes. Yet heating fungi for just 10 min at 70°C results in a >10-fold decrease in detectable RNA (see Table S3 at https://github.com/auchtung/Auchtung2018), suggesting that the contribution of fungi from cooked foods should be especially reduced. ITS2 cDNA analysis of controlled-diet stool samples did not identify additional fungal species (Fig. 2; see also Table S2I at https://github.com/auchtung/Auchtung2018); however, relative to ITS2 DNA, there was an increase in the abundance of *Candida* and the nonfungal microeukaryote *Blastocystis*, coupled with a decrease in the abundance of all other abundant taxa (Fig. 3). The detection of *Blastocystis* was unanticipated, as the most closely related *Blastocystis* sequences in GenBank have five mismatches (including the two 3′ nucleotides) with our reverse PCR primer, and suggests that *Blastocystis* RNA was present in an especially high abundance relative to fungal RNA.

To determine whether any fungi were not detected due to primer bias, we also amplified and sequenced the V6/V7 region of 18S rRNA genes from all stool samples (see Fig. S4 and Table S2J at https://github.com/auchtung/Auchtung2018). There was less diversity detected due to the greater sequence conservation of 18S rRNA genes than of ITS regions, and there were no additional fungal lineages detected via 18S rRNA genes that were not identified by ITS2 DNA analysis. However, three nonfungal microeukaryotes were identified which dominated stool samples from V1 (98.7% *Dientamoeba*) and V4 (91.1% *Blastocystis* and 8.9% *Entamoeba*).

To determine whether any fungi were undetected (despite being present in a sample) due to uneven nucleic acid extraction by a Mo Bio PowerMag microbiome (PMM) kit, DNA was also extracted from replicates of a subset of stool samples (all from V1) by two additional methods: a Mo Bio PowerSoil kit used by the HMP (19) and a protocol used by MetaHIT (20). Whereas ITS2 analysis of PowerSoil-extracted samples and 18S rRNA gene analysis of MetaHIT-extracted samples found no additional OTUs, ITS2 analysis of MetaHIT-extracted samples and 18S rRNA gene analysis of PowerSoil-extracted samples yielded four and two additional OTUs, respectively, that were not detected in the corresponding PMM-extracted samples (see Fig. S5 and S6 and Table S2K to P at https://github.com/auchtung/Auchtung2018). However, the additional OTUs were not abundant in V1 samples and were detected in PMM-extracted non-V1 samples, suggesting that their lack of detection here was due to chance rather than to a bias in the protocol.

As an additional test to examine whether any fungi were not detected by molecular analyses, and to gauge which fungi were able to survive gastrointestinal transit, stool samples were also plated on fungus-selective Sabouraud agar. Many of the most abundant fungal taxa identified by ITS2 sequencing of V1, V2, and V4 samples were also isolated by culturing (see Table S2Q at https://github.com/auchtung/Auchtung2018). Little was isolated from V3, in agreement with molecular analyses. C. albicans and *Saccharomyces* were the only fungi identified from plates grown anaerobically, and no fungal growth was detected on anoxic YCFA media (77), which can support the growth of most gut bacteria (21). In summary, there is no evidence that any fungi in stool were missed by biases in our approach to their detection; no fungi were consistently present in the gut unless they also existed in the saliva or diet.

### Examining fungal dynamics in gut-simulating bioreactors.

The GI tract may not be an accommodating environment for most fungi, as they must compete for nutrients while resisting inhibitory compounds produced by commensal bacteria such as short-chain fatty acids (22) and secondary bile acids (23). Fungal growth under the conditions found in the GI tract can be tested *in vitro* through the use of bioreactors, which are known to support growth of complex fecal bacterial communities (24). We examined the identity and abundance of fungi present within the complex communities of minibioreactor arrays after inoculation with stool from a healthy adult. Communities were cultivated at 37°C in media that varied in composition, under conditions of either an anoxic or hypoxic atmosphere (see Table S4 at https://github.com/auchtung/Auchtung2018). Samples were collected between days 2 and 7, when the communities typically had stabilized. Only one bioreactor, with high-sugar hypoxic media and a very low diversity of bacteria, contained elevated levels of fungi (≥10^4^ ITS2 copies/ng total DNA on days 4, 5, and 6). C. albicans was the only fungal species detected in these samples. Under conditions that resembled the healthy distal gut—low oxygen, simple sugars, and a diverse bacterial community—there was no evidence of fungal persistence.

### Assessing *Saccharomyces* stability in the gut in the absence of dietary input.

**FIG 4:**
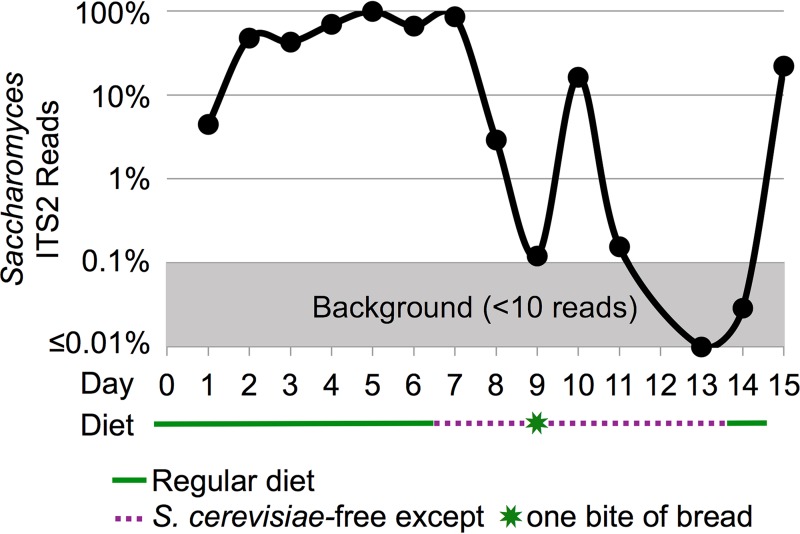
Relative ITS2 abundance of *Saccharomyces* in stool over time as a Saccharomyces cerevisiae-free diet was consumed. A logarithmically scaled chart showing the unrarefied relative abundances of *Saccharomyces* reads among ITS2 sequences amplified from stool DNA of a human volunteer is shown. Plant sequences were removed before analysis. The volunteer consumed a regular diet for 7 days and a Saccharomyces cerevisiae-free diet for 7 days and then resumed a regular diet. Day 8 data represent averages of results from two stool samples, and there was no sample on day 12.

We examined whether *Saccharomyces*, a genus of yeast that can survive gastrointestinal transit and one of the most commonly detected fungi in most examinations of human stool, would continue to be detected in a healthy adult volunteer after consumption of a S. cerevisiae-free diet (see Table S5A at https://github.com/auchtung/Auchtung2018). Although it has been shown that the probiotic S. cerevisiae var. boulardii is cleared within a few days of consumption (25), the retention of other strains of *Saccharomyces* has not been examined. Here, a volunteer collected all stool samples over the course of a 15-day experiment as follows: 1 week on a regular diet, followed by 1 week on a S. cerevisiae-free diet, followed by a final day where some foods containing S. cerevisiae were consumed. Samples were examined both by ITS2 and 18S rRNA gene analysis. Since there is only a single base pair in ITS2 distinguishing S. cerevisiae from related species (and since there are none in 18S rRNA genes), the OTU may contain multiple species of the genus and is referred to here as *Saccharomyces*. High levels of *Saccharomyces* were detected over the course of a normal diet (days 1 to 7), with the final sample containing 86% *Saccharomyces* ITS2 reads (Fig. 4; see also Table S5B at https://github.com/auchtung/Auchtung2018). After 2 days of consumption of a S. cerevisiae-free diet by the volunteer, *Saccharomyces* comprised just 0.1% of the reads (day 9). After unintentional consumption of a small amount of bread, there was a spike in the relative abundance of *Saccharomyces* on day 10 to 16% of the reads. However, by day 11 the abundance had again fallen to just 0.16% *Saccharomyces*. Reads of *Saccharomyces* were below the level of detection in the remaining S. cerevisiae-free diet samples (days 13 and 14). After the S. cerevisiae dietary exclusion had ended, *Saccharomyces* again became one of the most abundant taxa detected (day 15). The 18S rRNA gene data were similar to the ITS2 data, with the relative abundances of *Saccharomyces* just above the level of detection on days 9 and 11 and below the level of detection on days 13 and 14 (see Fig. S7 and Table S5C at https://github.com/auchtung/Auchtung2018). These data suggest that the *Saccharomyces* in the volunteer’s stool may have entirely originated from dietary S. cerevisiae.

### Tracing fluctuations in fecal Candida albicans to the oral cavity.

Candida albicans has long been studied for its role as an opportunistic pathogen (26). It is a leading cause of nosocomial infection, particularly in immunosuppressed patients (27). In our controlled-diet-experiment ITS2 analyses, it was the most abundant taxon overall in both stool and saliva. C. albicans is frequently described as colonizing the human GI tract; however, to our knowledge, there has been no study that has demonstrated that this is true in healthy human individuals.

**FIG 5:**
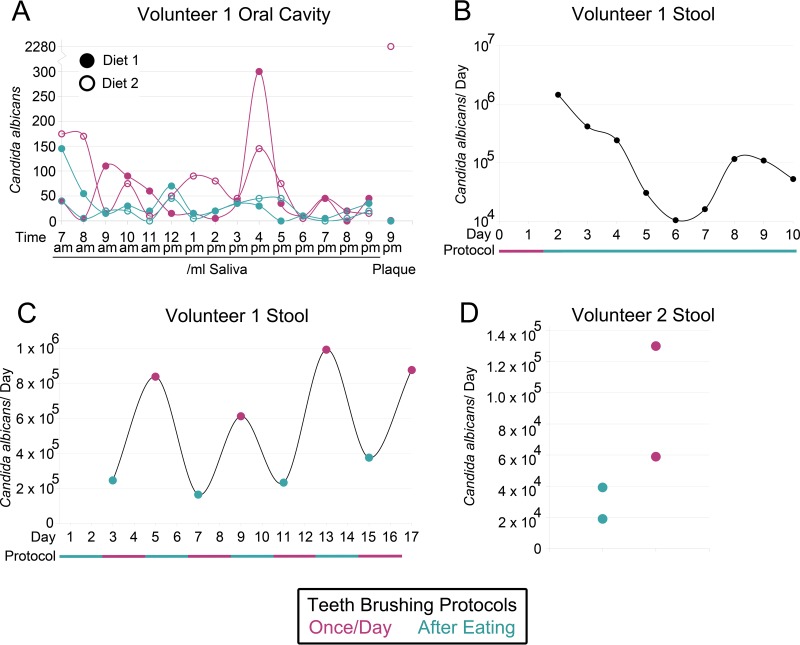
Candida albicans levels were decreased in the mouth and stool of healthy human volunteer(s) when teeth were brushed more often. (A) Concentrations of C. albicans in saliva throughout days when an adult volunteer either did or did not perform tooth brushing after eating. The volunteer consumed the same diet on both tooth-brushing protocols, the experiment was performed for two different diets, and levels of C. albicans were measured in a sample of plaque at the end of each day. (B) The total number of C. albicans cells in the stool of a volunteer over time (plotted on a log axis). Following a period of brushing teeth just once per day, the volunteer performed tooth brushing after every meal for 8 consecutive days. The diet was not the same from day to day but contained similar levels of sugars throughout the time period. (C and D) Lastly, the total number of C. albicans cells was measured in the stool of (C) a volunteer who alternately followed different tooth-brushing protocols for 2 days over the course of 16 days or of (D) a second volunteer who twice conducted each tooth-brushing protocol on nonconsecutive days. For all experiments, saliva, plaque, or stool was plated on Sabouraud plates containing antibiotics. Possible C. albicans colonies were later spotted on chromogenic media to distinguish the C. albicans colonies from closely related species and to adjust C. albicans numbers.

Since C. albicans is common in the oral cavity (15) and contributes to the formation of dental plaque and the development of caries (28), we wanted to assess the contribution of C. albicans in swallowed saliva to what is detected in stool. For all experiments, saliva or blended stool was plated on Sabouraud plates containing antibiotics. Possible C. albicans colonies were later spotted on chromogenic media to distinguish from closely related species. First, we confirmed that there was a difference in the levels of C. albicans in the saliva of a volunteer when the volunteer's teeth were brushed with conventional fluoride toothpaste either once per day (at night) or after every meal (3 to 4 times/day). Toothpaste helps remove plaque but does not significantly impact the viability of C. albicans (29). The levels of C. albicans measured in the oral cavity were relatively variable but were higher on average in saliva and plaque when teeth were brushed just once per day (Fig. 5A). Next, we monitored the change in abundance of C. albicans in stool when teeth were cleaned after every meal instead of once per day and saw that levels decreased from 10^6^ cells to 10^4^ to 10^5^ cells (Fig. 5B). Finally, to confirm that the decrease was not due to unrelated artifacts, we monitored C. albicans in stool while the protocols for cleaning of teeth were alternated: 2 days cleaning after every meal, followed by 2 days cleaning once per day, each done four times in total over 16 days. The total number of C. albicans colonies found in stool was significantly greater (two-sample equal variance *t* test *P* = 0.0007) after the volunteer brushed just once per day (mean = 8.3 × 10^5^) than after the volunteer brushed after every meal (mean = 2.5 × 10^5^) (Fig. 5C). A second volunteer who repeated a shorter version of the experiment on nonconsecutive days also had less C. albicans in stool after the volunteer brushed their teeth after every meal (Fig. 5D). Together, these experiments suggest that a large portion of C. albicans in the stool of the volunteers was derived from the oral cavity.

## DISCUSSION

Here we investigated fungal colonization of the healthy adult GI tract from multiple perspectives. We conducted a controlled-diet experiment to examine the abundance and identity of fungi present across multiple diets and then further investigated the dynamics of the two most abundant fungi, *Saccharomyces* and C. albicans, when dietary or oral hygiene practices were altered. We also examined fungi in bioreactors and in a large data set of healthy adults. From all avenues of investigation, the evidence suggests that fungi may not readily colonize the GI tracts of healthy adults in Western society.

Although some bacteria have been demonstrated to be consistently present at low levels in the GI tract (30), there was no evidence that that was the situation for any fungi not derived from food or the oral cavity. Quantitative PCR analysis of controlled-diet-experiment stool samples demonstrated that ITS2 were on average 3,000× less abundant than 16S rRNA genes. Considering that many of the ITS2 amplicons were from plants and that fungi often have higher ribosomal operon copy numbers than bacteria (31, 32), the fungi in our samples were at extremely low abundance (~0.001% to 0.01% of the population). This level of fungi is consistent with HMP metagenomic data (1); although early studies found fungal levels in adult stool that were ~10× higher (8, 9), the levels may have been overestimated due to extensive bacterial contamination of fungal sequence databases.

Despite the volunteers having consumed the same controlled diets, the fungi represented in volunteers’ stool DNA largely did not match by diet, due to the low fungal content of the control foods, the variable composition of the volunteers’ oral fungi, and the contribution of residual fungi from the volunteers’ regular diets. Only four taxa were present in at least one sample replicate of every diet of a volunteer (Candida albicans [V1, V2, and V3], *Saccharomyces* [V2], Aspergillus niger [V4], and *Penicillium* sp. [V1]). Both A. niger and *Penicillium* sp. were linked to the regular diets of individual volunteers and either were not detectable in the cDNA (A. niger) or were not detectable in multiple other samples from the same volunteer in the S. cerevisiae-free diet experiment (*Penicillium* sp.). This suggests there is little or no A. niger and *Penicillium* colonization of the GI tracts of the volunteers. *Saccharomyces* and C. albicans could have been from food and the mouth, respectively; however, to be certain of the source, we examined these fungi further. When a healthy adult volunteer abstained from consumption of food containing S. cerevisiae, the *Saccharomyces* OTU dropped below the limit of detection within a few days, suggesting that there had been little or no colonization. *Malassezia*, another fungal genus commonly detected in some studies (1, 13) and the only fungal genus other than *Saccharomyces* to be detected in all three longitudinal samples of multiple HMP volunteers, was not consistently detected in any volunteer’s controlled-diet stool samples by any of our molecular approaches, despite being present in the saliva of all volunteers. *Malassezia* may contaminate oral and stool samples due to high abundance on skin and may not be as well adapted for surviving passage through the GI tract.

C. albicans and related species have frequently been stated to colonize the GI tract (33, 34), though all evidence seems to be from their presence in human stool samples and from studies of inbred, typically antibiotic-treated animals. However, since the average person is thought to swallow over a liter of saliva/day (35), the GI tract is constantly dosed with oral microorganisms, including, for most people, species of *Candida* (15). Here, we showed that when a healthy adult volunteer increased the frequency of cleaning of teeth, the abundance of C. albicans in stool was lowered 10-fold to 100-fold. This suggests that the oral cavity may be the primary source of C. albicans detected in the stool of healthy people. Indeed, the fungal component of dental plaque of at least one cohort has been shown to be dominated by C. albicans (36). Taking additional measures to further reduce oral C. albicans levels, such as extra flossing and attention to elimination of plaque and reduction of consumption of refined sugars, may reduce levels of C. albicans in stool even further. This may explain why Hoffmann et al. (37) observed a positive correlation between fecal *Candida* levels and the consumption of carbohydrates. C. albicans is also much more common in Western populations, where refined sugar consumption is high (38, 39). Healthy mice are usually resistant to colonization by C. albicans (40); however, they are not typically maintained on a diet containing the simple sugars that encourage the growth of C. albicans. For one study where mice were fed a nutritionally adequate diet that included 10% sucrose, the mice had a sustained level of C. albicans in their feces (41). However, the oral cavities of the mice were not examined, so the role of that site in contributing to the C. albicans population is uncertain. Some work has been done investigating the effects of certain dietary compounds on C. albicans (42, 43), but more studies on the oral ecology of this opportunistic pathogen are needed. In bioreactors designed to mimic *in vivo* conditions in parts of the GI tract, we saw that C. albicans grew only when oxygen and a relatively high concentration of simple sugars were present. These conditions are relevant to the oral cavity and upper gastrointestinal tract, but not to the colon, where most bacteria are found. The bacterial community in the C. albicans growth-supporting bioreactors was also relatively simple, unlike that of a healthy GI tract, and therefore might have presented less resistance to fungal growth. It is possible that C. albicans colonizes both the mouth and the upper GI tract; in this regard, however, C. albicans may be akin to the oral bacteria that exacerbate disease when they colonize the gut (44, 45). Just as the Centers for Disease Control and Prevention recommends maintaining good oral health for preventing mouth, throat, or esophagus candidiasis (https://www.cdc.gov/fungal/diseases/candidiasis/thrush/), patients suffering from or at risk for developing a gastrointestinal disease might also benefit from increasing the attention that they pay to dental hygiene.

We conducted multiple analyses to ensure that fungi that might be colonizing the gastrointestinal tracts of volunteers were not missed by ITS2 sequencing of DNA. ITS2 from cDNA, 18S rRNA genes from DNA, ITS2 and 18S rRNA genes from DNA extracted by alternate methods, and fungi that grew on agar plates were also analyzed. No additional fungal species were detected by ITS2 cDNA and 18S rRNA gene analysis, though both approaches did reveal the presence of nonfungal microeukaryotes. ITS2 cDNA analysis specifically detected only high levels of C. albicans and the nonfungal microeukaryote *Blastocystis*, a known colonizer of the GI tract (46). The two alternative DNA extraction methods tested did not reveal any problems with detecting fungi in DNA extracted by our standard protocol. Additionally, most fungi isolated in culture grew only on plates of rich media in the presence of oxygen. There was no fungal growth on an intestine-like medium which is able to support the growth of nearly every bacterial species detected in stool by 16S rRNA gene analysis (21). In summary, the additional analyses failed to find deficiencies in our approach to fungal detection.

Analysis of the accumulation of 16S rRNA gene and ITS2 OTUs detected in healthy adult HMP stool samples demonstrated that while bacterial populations are similar from one individual to another and community sampling approaches saturation, fungal populations are more variable, and the community continues a steady rate of revealing new representatives as more samples are examined. In fact, in the entire cohort of 100 individuals, there were more ITS2 OTUs than 16S rRNA gene OTUs. This may, in part, have been because unique food-derived bacteria are hard to detect among the members of the enormous endemic bacterial population. An example is the genus *Lactobacillus*; once perceived to be autochthonous members of the GI tract, most species are actually derived from the mouth, from food, or from the upper GI tract (47). Whereas *Lactobacillus* sequences represent a minor fraction of the 16S rRNA gene data from stool, low-abundance fungal species from food and the oral cavity would be the predominant ITS2 OTUs detected under conditions in which there had been little or no fungal colonization of the GI tract.

We have presented multiple lines of evidence supporting the hypothesis that there are few or no fungal species indigenous to or colonizing the GI tracts of healthy adults in Western society. There are many possible reasons why gut-colonizing fungi might have been missed, but we consider them unlikely. They include the following possibilities. (i) Gut-specific strains might have been masked by other closely related fungi—at least for C. albicans, the same strain has been found in different parts of the body (48). (ii) Disease-free gut fungus-colonized individuals might have been missed due to our having examined only four volunteers. (iii) Fungi growing in the GI tract might have been degraded to the extent that they became completely undetectable in stool—even free DNA is known to survive transit (49). (iv) Fungi might colonize in extremely low numbers and bloom in the GI tract under healthy conditions not examined. (v) Fungi might exist in close association with host cells such that very few fungi are shed with fecal material. However, there are many reasons why fungi might not colonize, including inadequate physiological machinery for success in the environment, defenses of the mature human immune system, and a loss of gastrointestinal microbial diversity through recent or long-term evolution or exposures.

Many of the fungi detected by molecular analyses are not optimized for growth at 37°C (50, 51) and lack the genes and physiological traits reflective of an adaptation to the environment of the GI tract. Although some fungi such as C. albicans are able to survive the healthy adult GI tract, in order to colonize it, they face intense bacterial competition for nutrients and the threat of challenge by antimicrobials not present in the oral cavity, such as reactive nitrogen intermediates (52), peptides (53), and secondary bile acids (23). Physiologically relevant concentrations of short-chain fatty acids can also be fungistatic (22). Notably, the gut environment is not fully established in young children, where the immune system and competing bacterial communities are still developing. Though more data are needed, some studies have shown increased fungal diversity in children (54, 55).

Fungi may not persist in the GI tracts of those with a healthy immune system, and conversely, fungal colonization may be a symptom of illness. Those with a suppressed immune system are particularly susceptible to fungal invasion (56); the immune response mediated via multiple pattern recognition receptor families is important in the defense against fungi (57, 58). Furthermore, there are a multitude of studies showing fungal differences between healthy individuals and sick patients. However, there is a difference between detection of a microbe and proving that it colonizes that location—since the sick may spend more time indoors and/or eat differently, they could be exposed to different fungi. Therefore, it is necessary to consider confounding lifestyle and dietary differences to know whether any fungal gut colonizers are associated with the disease. If fungal colonization of the gut is associated only with disease, however, it might be beneficial to treat more patients with antifungals, especially if side effects can be avoided. In addition, the detection of increased levels of gastrointestinal fungi could alert clinicians to undiagnosed disorders.

Any indigenous or colonizing fungi might have disappeared as human diet and society has evolved. With modernization have come activities that limit microbial survival, transmission, and exposure, resulting in a loss of bacterial diversity and helminths from the GI tracts of humans (59, 60). There have been few microbiome studies conducted on non-Western communities, and most did not examine fungi. In the stool sample data set generated by Yatsunenko et al. (8), we found that subjects from Malawi and Venezuela had an increased proportion of fungal reads and an increased incidence of having any fungi compared to subjects from the United States (see Text S1 at https://github.com/auchtung/Auchtung2018). Yet, with the low overall abundance of fungi in human guts, deeper sequencing is needed to confidently determine whether there are increased levels of fungi in non-Western populations. In addition, measurements of oral and dietary fungi are needed to assess whether differences in stool fungi are due to increased levels of transient fungi or to genuine GI tract colonization.

It is also possible that humans lost gut fungi when they diverged from chimpanzees, primates, or more distant relatives, corresponding to changes in our diet and GI tract morphology (61). It has been shown that humans have had a loss of bacterial and archaeal diversity relative to other apes (62, 63). However, there have been few studies on fungi in the GI tracts of animals. Examination of stool from domesticated dogs and a wild gorilla found high fungal diversity (64, 65). Fungal populations in the guts of laboratory rodents show large variation from study to study, and many of the detected species can be explained by the presence of fungi in their chow and bedding (2, 66–69) (see Text S2 at https://github.com/auchtung/Auchtung2018). Clearly, much more research on fungal communities in the GI tracts of wild animals is needed.

In summary, we have shown that fungi detected from the GI tracts of volunteers across multiple diets were at very low abundance, were only transiently present from the diet or oral cavity, and did not grow when placed in gut-like conditions. In addition, the levels of two common fungal species in stool were shown to be directly affected by altering the oral hygiene or the dietary consumption of the volunteers, and analyses of data from larger cohorts were consistent with the possibility that fungi may not colonize the guts of healthy adults in Western society. More studies are needed that are mindful of dietary and oral fungi and that examine the relative contributions of immunological defenses, fungal ecology, and human evolution to limiting fungal persistence in the healthy modern adult GI tract.

## MATERIALS AND METHODS

### Comparison of fungi with bacteria/archaea in HMP stool samples.

ITS2 and 16S rRNA gene sequences from the Human Microbiome Project are available at NCBI (BioProject PRJNA356769) and the Human Microbiome Project website (http://www.hmpdacc.org), respectively. There were 148 samples that had at least 1,000 plant-filtered ITS2 and 16S rRNA gene reads. The data sets were rarefied to 1,000 reads by the use of single_rarefaction.py (70). Rarefaction curves were calculated with mothur v1.36.1, which uses a resampling-without-replacement approach (71).

### Controlled-diet experiment.

Four healthy people (ages between 25 and 37), all born and primarily raised on different continents (but who were residing in Houston, TX, for the duration of the study), with diverse dietary habits, consumed four identical diets (see Table S2A and B at https://github.com/auchtung/Auchtung2018). The diets were composed of a variety of food groups, largely low in fungi (except diet D), and were kept simple to facilitate analysis of all components. Volunteers did not consume probiotics or vitamins and did not chew gum but were allowed coffee, tea, and salt, as long as those foods were submitted for fungal sequencing analysis. There was no limit to the amount of food consumed. The diets were consumed nonconsecutively and in no set order over the course of 1 month, April 2016. Volunteers had the option to consume a 100-mg Brilliant Blue Lake (FD&C Blue 1 dye) pill at the start of each diet to clarify via staining that stool samples were primarily resulting from the particular diet and were ready to be collected. Volunteers collected a stool sample (12 to 222 g; mean, 77 g) following the appearance of the particular diet (or dye) in their stool. Saliva samples (typically three/diet) were collected at time points that were at least 2 h apart and at least 1 h after eating to reduce the number of plant particles that might obscure the detection of fungi. All samples were frozen at −80°C until nucleic acid extraction.

Using a Mo Bio PowerMag microbiome kit (including the phenol step), nucleic acids were extracted from dietary components, saliva (200 μl), and total stool which had been blended in 2 vol of prereduced phosphate-buffered saline (PBS)–0.5 g/liter cysteine (50 μl). For V1, replicate stool samples were extracted using the MetaHIT (20) and Mo Bio, Inc., PowerSoil protocols (19). All stool samples used for PCR were sampled in triplicate. Nucleic acids were treated with either RNase A (Qiagen) or DNase I (New England Biolabs). Representative RNA samples were analyzed by spectrophotometry and visualized on an agarose gel to confirm purity and integrity. rRNA was reverse transcribed using a Maxima H Minus kit (Thermo Fisher) with primer-specific conversion using ITS4R. As a control, RNA sample replicates with no reverse transcriptase were processed concurrently; DNA was rarely amplified, and taxa abundant in test samples were not detected (see Table S2F at https://github.com/auchtung/Auchtung2018). The ITS2 region, 18S rRNA gene V6/V7, and 16S rRNA gene V4 were amplified with primers ITS3F-ITS4R, 1152F-1428R, and 515F-806R, respectively, and sequenced on an Illumina MiSeq platform; reads were merged, clustered, subjected to chimera filtration, and mapped against databases as described previously (1). Nucleic acid from each of the three stool samples was amplified for ITS2 DNA, ITS2 cDNA, and 18S rRNA genes, but only a single randomly chosen replicate was amplified for 16S rRNA gene analysis. Every sequenced region averaged ≥10,000 reads/sample. Because the maximum amount of sequencing barcode bleed-over seen in 42 control samples was 7 reads, an OTU was considered present if there were at least 10 reads detected in a sample. Three PBS/collection dish/blender controls yielded no isolates and <10 ITS2 DNA and cDNA reads (see Table S2F at https://github.com/auchtung/Auchtung2018). For blending, nucleic acid extraction, and PCR amplification, the order of sample processing was randomized. All primer sequences are listed in Table S8 at https://github.com/auchtung/Auchtung2018. Taxon box plots and stacked bar plots were generated in R (72), utilizing the phyloseq package (73).

Replicates of all blended stool samples (50 μl) were plated on Sabouraud agar (4% dextrose, 1% peptone, 100 μg/ml ampicillin, 50 μg/ml chloramphenicol) and grown at both room temperature and 37°C under conditions of ambient and anoxic (90% N_2_, 5% CO_2_, 5% H_2_) atmospheres. Colonies were identified by Sanger sequencing following PCR amplification with ITS3F-ITS4R and, where necessary, 18S rRNA gene-targeting 1152F-1428R or 16S rRNA gene-targeting 27F-907R (74). To look for fungi that grow under conditions more physiologically similar to those of the gut, frozen replicates of the uncontrolled-diet samples were later plated on YCFA media (77) containing 50 μg/ml chloramphenicol, 100 μg/ml ampicillin, 10 μg/ml tetracycline, and 50 μg/ml kanamycin with or without 250 μg/ml bovine bile and placed in an anoxic atmosphere for at least 2 weeks.

ITS2-specific primers ITS3F and ITS4R and 16S rRNA gene-specific primers Bact1369F and Prok1492R were used in quantitative PCR analysis of controlled-diet fecal DNA samples using Applied Biosystems Power SYBR green PCR master mix following the manufacturer's instructions, with 60 s (ITS2) or 30 s (16S rRNA gene) of annealing/extension at 60°C. To create nonsupercoiled standard curve templates (75), DNA from Malassezia cuniculi (450-bp ITS2) or Escherichia coli* rrsA* was amplified using the primers specified above, except that the procedure also used an additional 64 (ITS3F and ITS4R), 18 (Bact1369F), or 19 (Prok1492R) 5′ nucleotides (nt) so that quantitative PCR primers would not have to anneal to the very end of the template.

### Bioreactors.

Experiments were conducted in minibioreactor arrays as previously described (24) using five different media under conditions of either an anoxic (5% CO_2_, 95% N_2_) or hypoxic (2% O_2_, 5% CO_2_, 93% N_2_) atmosphere (see Table S4 at https://github.com/auchtung/Auchtung2018). For each medium/atmosphere condition, triplicate 15-ml reactors were inoculated with a 25% fecal slurry (containing 75% reduced PBS) and allowed to sit for 16 h (anoxic reactors) or 4 h (hypoxic reactors) before flow was initiated at 1.825 ml/h (anoxic) or 3.75 ml/h (hypoxic). The hypoxic bioreactors had a shorter initial wait and higher flow rate to more closely resemble the upper GI tract. Samples for DNA extraction (as described in reference 76) and sequencing were collected daily from each reactor over the course of 7 days. Quantitative PCR was performed as described above.

### *Saccharomyces* experiment.

A single healthy adult volunteer ate regularly for 1 week and then avoided eating foods containing S. cerevisiae for 1 week, followed by 1 day of eating foods with a large amount of S. cerevisiae (see Table S5A at https://github.com/auchtung/Auchtung2018). Over the course of the 15 days, the stool samples were collected and frozen, typically once daily. Two months later, samples were collected during 3 days of a regular diet. DNA from approximately 250 mg frozen stool was extracted using the Mo Bio PowerSoil protocol and analyzed as described above, with an average of ≥55,000 ITS2 and ≥25,000 18S rRNA gene reads/sample. We defined the limit of detection as 10 reads of *Saccharomyces*, since 27 pure culture controls had ≤10 reads from barcode bleed-over.

### Candida albicans experiments.

To test the effect of increased tooth brushing (after every meal, within 30 min) on C. albicans levels in saliva of a healthy adult volunteer, for every hour from 7 a.m. to 9 p.m. over 2 consecutive days during which the same diet was consumed, 200 μl of saliva was spread on Sabouraud media containing ampicillin and chloramphenicol. The numbers of colonies were counted after 24 h at 37°C and were adjusted after 48 h following verification of the presence of previously small colonies. Up to 10 colonies/plate were spotted onto chromogenic BBL CHROMagar Candida (BD) and grown for at least 48 h to distinguish C. albicans from related species. Since the cells do not grow as well on the chromogenic agar, resulting in fewer colonies, the medium was used only to assess the identity of Sabouraud-grown colonies. The reported number of C. albicans cells in each sample was corrected using these data.

To test how the levels of C. albicans in stool of healthy volunteers were affected by variations in tooth-brushing protocols, the following experiments were conducted. First, over the course of 8 days, during which time the volunteer brushed their teeth after every meal instead of once per day, entire stool samples were collected and blended with PBS, and 150 μl was plated in triplicate on Sabouraud media containing antibiotics. In a second experiment, the following protocol was repeated by the same volunteer four times: brushing of teeth once per day (at night) for 2 days and then after each meal for 2 days. For the course of the experiment, the volunteer defecated once per day at the same time (±30 min). On the day after each 2-day protocol, a full stool sample was collected and blended with PBS, and 50 μl was plated in triplicate. A variation of this experiment was conducted by a second volunteer but on nonconsecutive days and following each protocol twice. Volunteers were instructed to eat similar amounts of sugary foods between the protocols, avoid *Penicillium*-rich cheeses, and avoid heavy consumption of alcohol. At the end of the stool experiments, all presumed C. albicans colonies from at least one random replicate plate/day were spotted onto chromogenic agar as described above. The *t* test was performed in Excel.

### Ethics statement.

The Institutional Review Board of Baylor College of Medicine approved all study protocols. All participants provided written informed consent (protocols H-32521 and H-29645).

### Data availability.

Sequence data were deposited in NCBI's Sequence Read Archive (BioProject PRJNA379437) and GenBank (accession no. KY931930 to KY932673 and KY932725 to KY933059).
